# ADAR1-mediated RNA editing is a novel oncogenic process in thyroid cancer and regulates miR-200 activity

**DOI:** 10.1038/s41388-020-1248-x

**Published:** 2020-03-10

**Authors:** Julia Ramírez-Moya, Allison R. Baker, Frank J. Slack, Pilar Santisteban

**Affiliations:** 1 Instituto de Investigaciones Biomédicas “Alberto Sols”; Consejo Superior de Investigaciones Científicas (CSIC), Universidad Autónoma de Madrid (UAM), Madrid, Spain; 2 Department of Pathology, Harvard Medical School Initiative for RNA Medicine, Beth Israel Deaconess Medical Center, Harvard Medical School, Boston, MA USA; 3 Centro de Investigación Biomédica en Red de Cáncer (CIBERONC) Instituto de Salud Carlos III (ISCIII), Madrid, Spain

**Keywords:** Thyroid cancer, siRNAs

## Abstract

Adenosine deaminases acting on RNA (ADARs) convert adenosine to inosine in double-stranded RNA. A-to-I editing of RNA is a widespread posttranscriptional process that has recently emerged as an important mechanism in cancer biology. A-to-I editing levels are high in several human cancers, including thyroid cancer, but ADAR1 editase-dependent mechanisms governing thyroid cancer progression are unexplored. To address the importance of RNA A-to-I editing in thyroid cancer, we examined the role of *ADAR1*. Loss-of-function analysis showed that *ADAR1* suppression profoundly repressed proliferation, invasion, and migration in thyroid tumor cell models. These observations were validated in an in vivo xenograft model, which showed that *ADAR1*-silenced cells had a diminished ability to form tumors. RNA editing of miRNAs has the potential to markedly alter target recognition. According to TCGA data, the tumor suppressor miR-200b is overedited in thyroid tumors, and its levels of editing correlate with a worse progression-free survival and disease stage. We confirmed miR-200b overediting in thyroid tumors and we showed that edited miR-200b has weakened activity against its target gene *ZEB1* in thyroid cancer cells, likely explaining the reduced aggressiveness of *ADAR1*-silenced cells. We also found that RAS, but not BRAF, modulates ADAR1 levels, an effect mediated predominantly by PI3K and in part by MAPK. Lastly, pharmacological inhibition of ADAR1 activity with the editing inhibitor 8-azaadenosine reduced cancer cell aggressiveness. Overall, our data implicate ADAR1-mediated A-to-I editing as an important pathway in thyroid cancer progression, and highlight RNA editing as a potential therapeutic target in thyroid cancer.

## Introduction

Mutations in DNA are not the only source of modifications to the genomic content of human cells. RNA editing is a widespread posttranscriptional mechanism to engender genomic diversity by specifically and reproducibly changing selected RNA sequences without altering the genomic blueprint [1, 2]. The conversion of adenosine to inosine (A-to-I editing) is considered the most common RNA modification in mammals, with millions of editing sites detected thus far in humans [2, 3]. RNA editing is catalyzed by the adenosine deaminase acting on RNA (ADAR) family of enzymes in double-stranded RNA of more than 20 bp in length [4], formed both intra- and intermolecularly [5]. Among the three types of known mammalian ADARs, ADAR1 is the most prevalent and is expressed ubiquitously [6]. Transcription from independent promoters generates two isoforms of ADAR1—a full length interferon-inducible ADAR1 p150 transcript, and a shorter and constitutively expressed ADAR1 p110 form [7].

As a result of RNA editing, inosines are interpreted by the cellular machinery as guanosines, and are base-paired with cytosine, making the effect of A-to-I editing similar to an A-to-G substitution [5]. Accordingly, editing of coding mRNAs may lead to the creation of stop codons, or to changes in splice sites or missense codons that give rise to recoding, creating structurally and functionally unique isoforms of proteins from the same transcript. The majority of editing activity in human cells, however, occurs in repetitive element regions and noncoding sequences, modifying regulatory RNAs and their binding sites [1, 3, 5].

Some microRNA (miRNA) precursors undergo A-to-I editing, which regulates the expression and/or function of mature miRNAs. miRNAs are short noncoding RNAs that function posttranscriptionally to suppress gene expression through interactions of their seed region with complementary sequences in the 3′-untranslated regions (UTRs) of target messenger RNAs [8], and their misregulation can lead to human diseases such as cancer [8–12]. In some contexts, editing of a primary miRNA can affect its biogenesis, inhibiting miRNA maturation. Alternatively, it can alter the recognition of the target mRNA, particularly if editing occurs within the seed sequence of the miRNA [7].

Thus, A-to-I alterations result in dynamic RNA mutations, which could ultimately lead to cancer outcomes similar to those caused by genomic mutations, but with higher flexibility as they affect only a fraction of the transcripts. Aberrant RNA editing is an underexplored mechanism that can reproducibly alter protein and regulatory RNA sequences, acting as a driver of carcinogenesis, and therefore, a potential therapeutic target [13]. Indeed, A-to-I editing and the enzymes mediating this modification are significantly altered in cancer. In most tumor types, editing levels are elevated when compared with matched normal tissues, with the strongest levels reported in breast, thyroid, and head and neck cancers [13, 14]. By contrast, a significant number of RNA editing sites are underedited in kidney chromophobe and kidney papillary cell carcinoma tumors, among others [13, 15].

According to data from The Cancer Genome Atlas (TCGA), thyroid cancer shows high levels of editing when compared with matched normal tissue [13, 14], and should thus provide a good model to study the RNA editing process. Furthermore, although the mRNA expression of *ADAR1* may not directly reflect the editing activity [13, 16], its expression is low in thyroid carcinomas but is significantly elevated over normal tissue [13, 14], and high *ADAR1* mRNA expression correlates with worse progression-free survival [13]. Thyroid cancer is the most frequent endocrine malignancy and is the most rapidly increasing of all cancers in the United States [17]. Thyroid cancer generally has a good outcome [18], and thyroidectomy with radioiodine ablation and thyroid-stimulating hormone suppressive therapy remains the cornerstone of treatment for patients with thyroid cancer, although it is often not curative. Indeed, some patients develop aggressive forms of the disease that are untreatable and the molecular bases are poorly understood [18]. Accordingly, a better understanding of thyroid cancer is essential for the development of new effective therapies. The classical view of thyroid cancer pathogenesis considers thyroid carcinomas as tumors accumulating mutations that drive progression through a dedifferentiation process, giving rise initially to well-differentiated carcinomas such as papillary (PTC) and follicular (FTC), and progressing to poorly differentiated (PDTC) and undifferentiated or anaplastic (ATC) thyroid carcinoma [18]. Recently, a molecular classification of thyroid carcinomas based on mutations in the main known signaling pathways, MAPK, and PI3K, has been established. Further, two genetic types of carcinomas have been defined based on the manner in which the oncogenes *BRAF* and *RAS* promote tumor initiation and progression, and their relationship to the main pathways [19]. *BRAF*-driven tumors have high MAPK activity, whereas *RAS*-driven tumors largely show a hyperactivation of the PI3K pathway [20, 21]. Nevertheless, to date no studies have addressed the relationship between ADAR1-dependent A-to-I editing and the main oncogenic pathways and driver mutations in thyroid cancer.

Here, we establish, for the first time to our knowledge, the functional consequences of A-to-I editing in thyroid cancer cells. We show that *ADAR1* expression and consequent RNA editing alters thyroid cancer cell aggressiveness through its effects on proliferation, invasion, migration, and 3D growth in vitro, and tumor growth in vivo. We explored the molecular mechanisms underlying these effects, finding that the tumor suppressor miR-200b is overedited in thyroid tumors, and that RNA editing impairs its ability to inhibit the epithelial–mesenchymal transition (EMT) marker ZEB1. Finally, we relate the main thyroid cancer signaling pathways to ADAR1 isoform levels, and we provide evidence that pharmacological inhibition of A-to-I editing in thyroid cancer cells diminishes aggressiveness in vitro, highlighting RNA editing as an exciting subject for research into thyroid cancer mechanisms and treatment options.

## Results

### *ADAR1* silencing diminishes thyroid cancer cell aggressiveness in vitro and in vivo

**Fig. 1:**
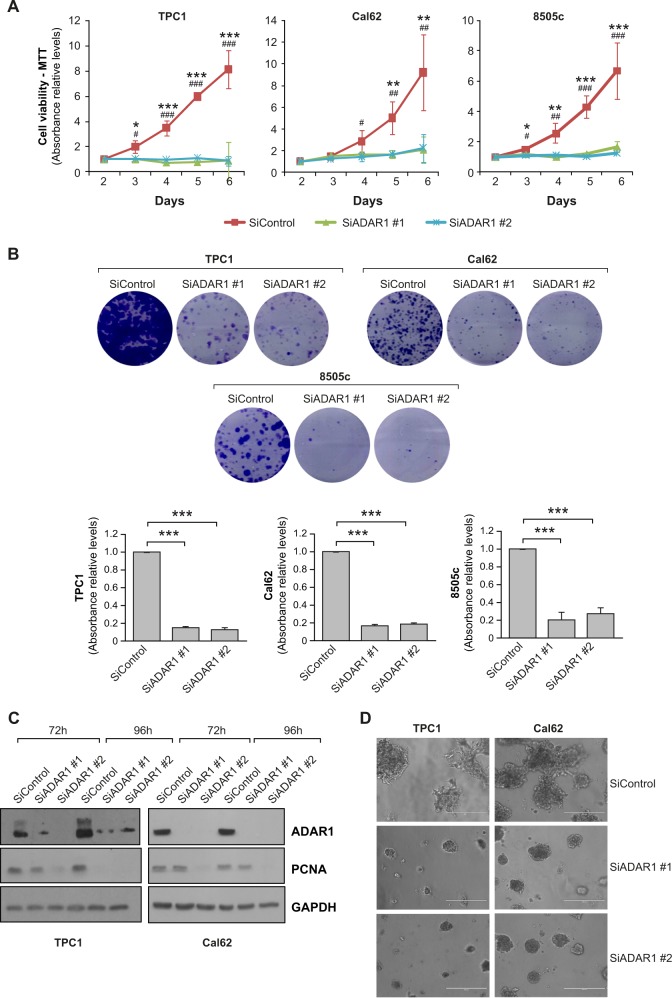
*ADAR1* knockdown reduces thyroid cancer cells proliferation and 3D growth. TPC1, Cal62, and 8505c cell lines were transfected with two different siRNAs against *ADAR1* (siADAR1 #1 and siADAR1 #2) or a control siRNA (siControl). **a** MTT assay at the indicated time points. **b** Upper panel: representative images of crystal violet-stained colonies. Bottom panel: quantification of crystal violet absorbance. **c** Immunoblot of ADAR1 and proliferating cell nuclear antigen (PCNA) at 72 and 96 h after transfection. GAPDH was used as a loading control. **d** 3D cell culture. Values represent mean ± SD (*n* = 3). Asterisks represents significant difference between siADAR1 #1 and siControl, and Hash represents significant difference between siADAR #2 the siControl. *^/^^#^*p* < 0.05; **^/^^##^*p* < 0.01; ***^/^^###^*p* < 0.001.

**Fig. 2:**
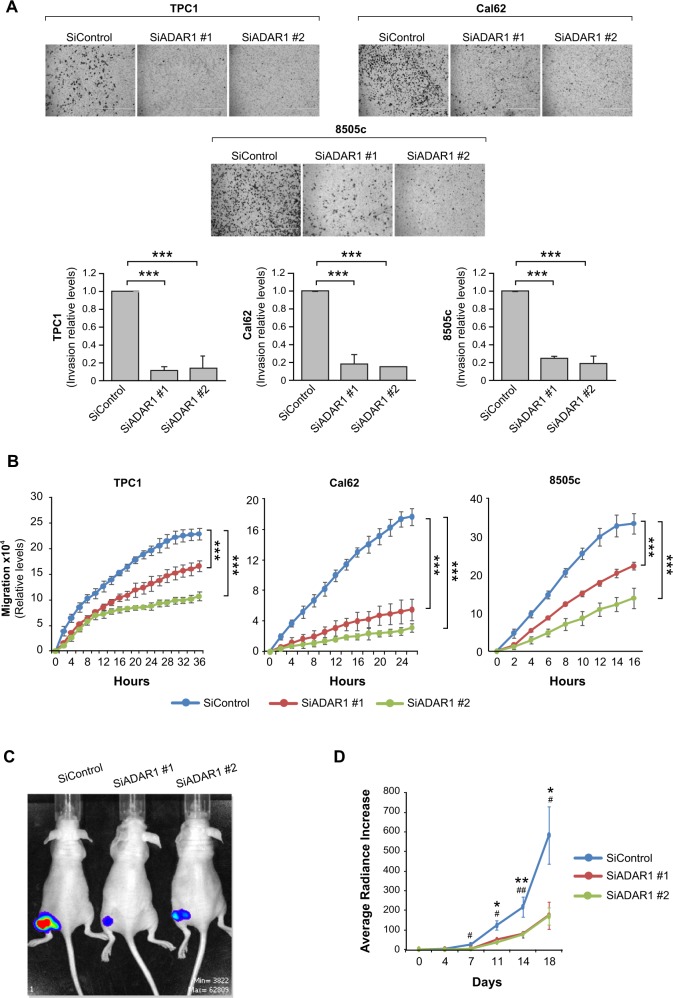
*ADAR1* knockdown reduces thyroid cells invasion, migration in vitro and xenograft tumor growth in vivo. TPC1, Cal62, and 8505c cell lines were transfected with two different siRNAs against *ADAR1* (siADAR1 #1 and siADAR1 #2) or a control siRNA (siControl). **a** Quantification of cell invasion. Upper panel: representative images of the lower chamber (invading cells). Bottom panel: cell invasion relative to siControl cells. **b** Quantification from a wound-healing assay at the indicated time points after scratching. **c**, **d** Xenograft tumors were generated by subcutaneous injection with Cal62-Luc cells transfected previously with siControl (*n* = 8), siADAR1 #1 (*n* = 7), or siADAR1 #2 (*n* = 7). **c** Endpoint (day 18) bioluminescent signal of the generated tumors. **d** Tumor radiance quantification at the indicated time points. Values represent mean ± SD (*n* = 3). ***p < 0.001 for the in vitro techniques (A and B), and mean ± SEM. *^/^^#^*p* < 0.05; **^/##^*p* < 0.01 for the in vivo experiment (**d**).

*ADAR1* expression is slightly higher in thyroid tumors than in matched normal samples [13–15]. However, according to TCGA data (https://portal.gdc.cancer.gov/), more robust differences are found in the levels of RNA editing, with thyroid tumors showing one of the highest overediting levels when compared with matched normal tissue [13–15]. Thyroid tumor cells thus provide a novel model to study the effect of A-to-I editing. To test the importance of ADAR1 in thyroid cancer, we performed *ADAR1* loss-of-function assays in three thyroid cancer cell lines: a PTC cell line (TPC1) and two ATC cell lines (Cal62 and 8505c). We used two different siRNAs targeting *ADAR1*, finding that both markedly decreased *ADAR1* mRNA levels (Fig. S1a), which was accompanied by a corresponding loss in A-to-I editing activity (Fig. S1b). *ADAR1* silencing profoundly suppressed cell viability and proliferation measured by MTT reduction and crystal violet staining, and decreased the levels of the proliferation marker PCNA (Fig. 1a–c). We confirmed these observations using a three-dimensional (3D) model, which better mimics the complexity and heterogeneity of tumors [22]. *ADAR1* knockdown reduced the growth of TPC1 and Cal62 cells in 3D Matrigel cultures (Fig. 1d). Of note, we observed that in contrast to control cells, which invaded the 3D Matrigel substrate and ultimately attached to the bottom of the plate, silenced cells were unable to invade and remained as spheres over time (Fig. 1d). As expected, quantification of invasion (Fig. 2a) and migration (Fig. 2b) capacity revealed a marked decrease in both parameters in all *ADAR1*-silenced cells as compared with control cells.

We next used a heterotopic xenograft model utilizing luciferase-expressing Cal62-Luc cells to question whether the repression of *ADAR1* expression in aggressive thyroid cells affects tumor growth in vivo. As anticipated, tumor growth was significantly delayed in the *ADAR1*-silenced group (Fig. 2c, d and Fig. S2a, b). No differences were noted between the weight of the mice injected with *ADAR1*-silenced or control cells, suggesting no adverse effects of the silenced cells (Fig. S2c).

### *ADAR1* silencing reduces ZEB1 levels by abolishing editing of miR-200b

**Fig. 3:**
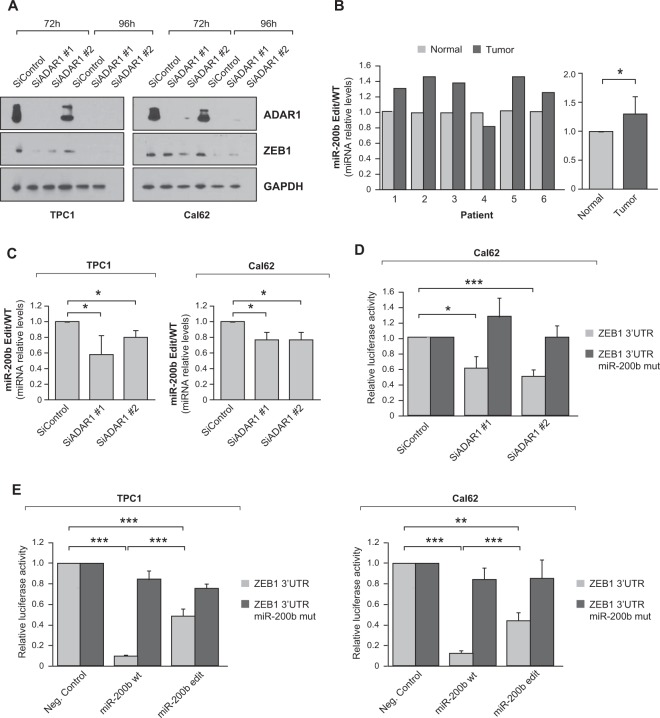
The ADAR1-dependent editing in miR-200b, increased in thyroid cancer patients, decreasing its binding to ZEB1 3′UTR. **a** Immunoblot for ADAR1 and ZEB1 72 and 92 h after TPC1 and Cal62 siControl, siADAR1 #1 or siADAR1 #2 transfection. GAPDH was used as a loading control. **b** Left: relative miR-200b edited (Edit)/wild-type (WT) miRNA levels in six PTC patients (contralateral and normal thyroid tissue). Right: total average of miR-200b Edit/WT miRNA relative levels. **c** miR-200b Edit/WT miRNA relative levels in TPC1 and Cal62 cells transfected with the siRNAs siControl, siADAR1 #1 or siADAR1 #2. **d** *Renilla* luciferase reporter activity relative to Firefly luciferase was evaluated 72 h after transfection of *ZEB1* 3′UTR or the same construction with mutated predicted miR-200b-3p binding sites (*ZEB1* 3′UTR miR-200b mut) in Cal62 cells co-transfected with the siRNAs siControl, siADAR1 #1 or siADAR1 #2. **e** *Renilla* luciferase reporter activity relative to Firefly luciferase was evaluated 72 h after transfection of *ZEB1* 3′UTR or the same construction with mutated predicted miR-200b-3p binding sites (*ZEB1* 3′UTR miR-200b mut) in TPC1 and Cal62 cells co-transfected with the WT miR-200b or edited (edit) miR-200b mimics or a negative control (Neg.Control). Values represent mean ± SD (*n* = 3). *p < 0.05; **p < 0.01; ***p < 0.001.

Given that *ADAR1* silencing suppresses thyroid cancer cell aggressiveness, we next examined the effects of *ADAR1* silencing on EMT, a cancer hallmark involved in thyroid cancer progression. We tested the expression of several EMT regulators (not shown) and found that ZEB1 (zinc finger E-box-binding homeobox 1) was the most dysregulated upon *ADAR1* silencing (Fig. 3a). ZEB1 is a critical activator of EMT in thyroid [23, 24] and other cancer [25–29] types. Silencing of *ADAR1* led to a corresponding decrease in the abundance of ZEB1 in both TPC1 and Cal62 cells (Fig. 3a). ZEB1 is regulated in different tissues by miR-200b-3p [30–32], which directly targets the *ZEB1* 3′UTR and reduces its expression. Accordingly, miR-200b has been described as a tumor suppressor miRNA that disrupts EMT programs [33, 34], and it is under intensive clinical investigation as a promising therapeutic cancer target [35]. Although relatively little is known about miR-200b in thyroid cancer, it is downregulated in ATC, the most aggressive form of the disease [36, 37]. It has been recently reported that a recurrent editing site occurs in the seed region of miR-200b-3p in multiple cancers in TCGA cohort, including thyroid cancer where miR-200b is considerably overedited [38]. Of note, the editing hotspot in miR-200b is significantly associated with disease stage, and a worse progression-free survival [38]. We validated miR-200b overediting in six thyroid tumors and their contralateral normal tissue (clinical characteristics are summarized in Table S1) by qRT-PCR, confirming that the miR-200b edited/wild-type (WT) ratio is significantly increased in tumor samples (Fig. 3b).

To test whether abnormal expression of *ADAR1* in thyroid cancer cell lines affects miR-200b editing, we analyzed the miR-200b edited/WT ratio in *ADAR1*-silenced TPC1 and Cal62 cells, and found that the editing ratio decreased (Fig. 3c). This result indicates that ADAR1 contributes to miR-200b editing, and points to its participation in miR-200b editing in tumor thyroid cells. To question whether the decrease in ZEB1 expression in *ADAR1*-silenced cells was due to miR-200b-mediated repression, we performed luciferase reporter assays in control and *ADAR1*-silenced cells to test the *ZEB1* 3′UTRs containing a WT or a mutated (negative control) miR-200b binding sites. The *Renilla*/Firefly ratio was reduced in *ADAR1*-silenced cells with WT *ZEB1* 3′UTR, confirming that the decrease in ZEB1 expression was mediated through its 3′UTR. By contrast, the *Renilla*/Firefly ratio was unchanged when expressed from the mutated form of the 3′UTR (Fig. 3d), supporting that loss of ZEB1 expression was due to the miR-200b binding sites within its 3′UTR. Because the editing hotspot in miR-200b is located in its seed region [38], we hypothesized that the under-editing of miR-200b provoked by *ADAR1* silencing would lead to high levels of WT miR-200b, which has a higher affinity for the *ZEB1* 3′UTR, leading to the suppression of ZEB1 expression. To test this, we assessed the activity of WT and edited miR-200b on the *ZEB1* 3′UTR, and their ability to inhibit ZEB1 expression, and hence invasion capacity, in thyroid cancer cells.

We found that WT miR-200b directly represses *ZEB1* through its 3′UTR in TPC1 and Cal62 thyroid cancer cells, as its overexpression significantly decreased the activity of a co-transfected luciferase reporter construct containing the putative miR-200b binding region in the *ZEB1* 3′UTR. Conversely, nonsignificant changes in luciferase activity were observed when the *ZEB1* 3′UTR construct was mutated in the predicted binding sites for miR-200b (Fig. 3e). When we repeated the assays with the edited miR-200b mimic, we found a significant decrease in its ability to repress luciferase activity through the *ZEB1* 3′UTR in TPC1 and Cal62 cell lines (Fig. 3e).

**Fig. 4:**
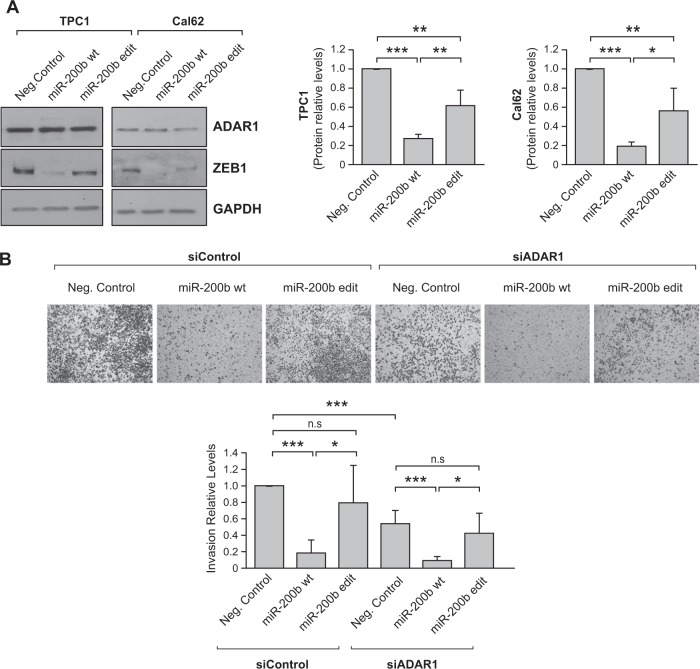
Editing of miR-200b impairs its ability to inhibit ZEB1, modulating the thyroid cell invasion capacity. **a** Left: representative immunoblot for ADAR1 and ZEB1 72 h after transfection with WT miR-200b or edited (edit) miR-200b mimics or negative control in TPC1 and Cal62 cells. GAPDH was used as a loading control. Right: quantification relative to the negative control transfected cells. **b** Cal62 cells were co-transfected with an siRNA (siControl or siADAR1) and an miRNA mimic (negative control, WT miR-200b wt or edit miR-200b mimics). Upper panel: representative images of the lower chamber (invading cells). Bottom panel: quantification of invasion relative to siControl—Neg. Control cells. Values represent mean ± SD (*n* = 3). *p < 0.05; **p < 0.01; ***p < 0.001, ns nonsignificant.

We performed western blotting to confirm these data. Accordingly, ZEB1 protein expression was markedly lower in both TPC1 and Cal62 cells transfected with the WT miR-200b mimic than in control cells, whereas transfection with the edited miR-200b mimic resulted in an intermediate level of ZEB1 (Fig. 4a). Interestingly, miR-200b (WT or the edited form) do not affect ADAR1 protein expression levels (Fig. 4a). Finally, as the miR-200b-ZEB1 axis is mainly involved in EMT and invasion, we performed Matrigel invasion assays in control and *ADAR1*-silenced Cal62 cells overexpressing the WT or edited miR-200b. As expected, expression of WT miR-200b decreased invasion in control and ADAR1-silenced cells, whereas expression of edited miR-200b had no effect (Fig. 4b), likely due to its reduced capacity to inhibit ZEB1.

### ADAR1 regulation in thyroid cancer cells is PI3K-dependent

**Fig. 5:**
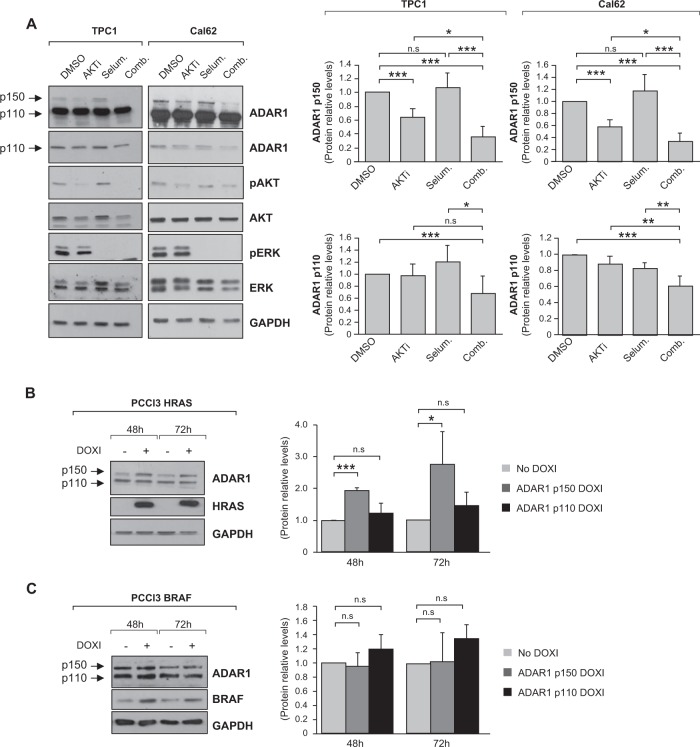
ADAR1 regulation in thyroid cancer. **a** Cal62 and TPC1 cells were treated with DMSO (vehicle), 10 µM AKTi, 50 µM selumetinib or the combination of both inhibitors. Left: representative immunoblot for the indicated proteins. GAPDH was used as a loading control. Right: ADAR1 p150 (upped panel) and ADAR1 p110 (bottom panel) quantification relative to DMSO-treated cells. PCCl3-inducible HRAS (**b**) or BRAF (**c**) cells were treated with doxycycline for 48 and 72 h. Left panels: representative immunoblots. GAPDH was used as a loading control. Right panels: ADAR1 p150 and ADAR1 p110 protein level quantification relative to nontreated cells. Values represent mean ± SD (*n* = 3). *p < 0.05; **p < 0.01; ***p < 0.001, ns nonsignificant.

We next focused on the regulation of ADAR1 in thyroid cancer. As mentioned earlier, PI3K and MAPK are the two main signaling pathways involved in thyroid cancer development and progression [19–21]. To block these pathways in tumor cells, we used the inhibitors AKTi and selumetinib, or their combination. Assays were performed in TPC1 cells, which are mutated in *RET/PTC*, and in Cal62 cells, which are mutant for *KRAS*. Inhibition of the PI3K pathway significantly decreased the expression of the ADAR1 p150 isoform in both cell lines (Fig. 5a). In addition, although selumetinib alone had no effect on ADAR1 expression, the combination of both inhibitors inhibited both ADAR1 isoforms (Fig. 5a).

We next investigated whether ADAR1 expression is altered during the earliest steps of thyroid tumorigenesis driven by RAS and BRAF, the two main oncogenes in thyroid cancer [19]. To do this, we used rat thyroid-derived PCCl3 cells conditionally expressing HRAS^V12^ (PCCl3-HRAS) or BRAF^V600E^ (PCCl3-BRAF) upon addition of doxycycline. Doxycycline treatment for 48 and 72 h increased ADAR1 p150 isoform levels in PCCl3-HRAS cells (Fig. 5b) but not in PC-BRAF cells (Fig. 5c), indicating that HRAS, but likely not BRAF, induces ADAR1 p150 expression. By contrast, no changes were detected in the levels of the ADAR1 p110 isoform upon activation of RAS or BRAF.

### 8-azaadenosine blocks RNA editing and inhibits proliferation, 3D growth, invasion, and migration in thyroid cancer cells

**Fig. 6:**
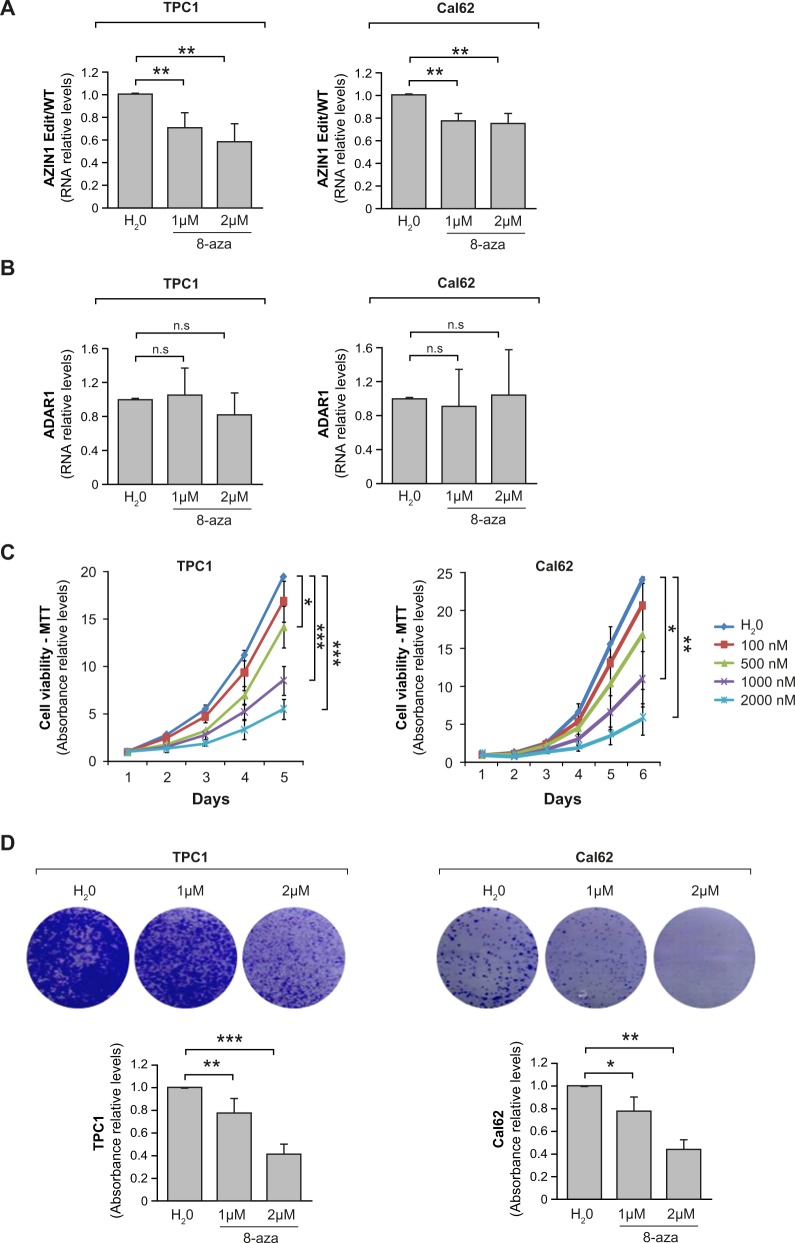
Inhibition of RNA A-to-I editing reduces viability and proliferation of thyroid cancer cells. TPC1 and Cal62 cells were treated with 8-azaadenosine (8-aza) (1 µM or 2 µM) or water (H_2_0). **a** Relative *AZIN1* edited/*AZIN1* wild-type (*AZIN1* Edit/WT) mRNA levels assayed by RESS-qRT-PCR 72 h post treatment. **b** Relative *ADAR1* mRNA level assayed by qRT-PCR 72 h post treatment. **c** MTT assays at the indicated time points at 100 nM, 500 nM, 1 µM, and 2 µM 8-aza concentration. **d** Upper panel: representative images of crystal violet-stained cells. Bottom panel: quantification relative to nontreated cells. Values represent mean ± SD (*n* = 3). *p < 0.05; **p < 0.01; ***p < 0.001.

**Fig. 7:**
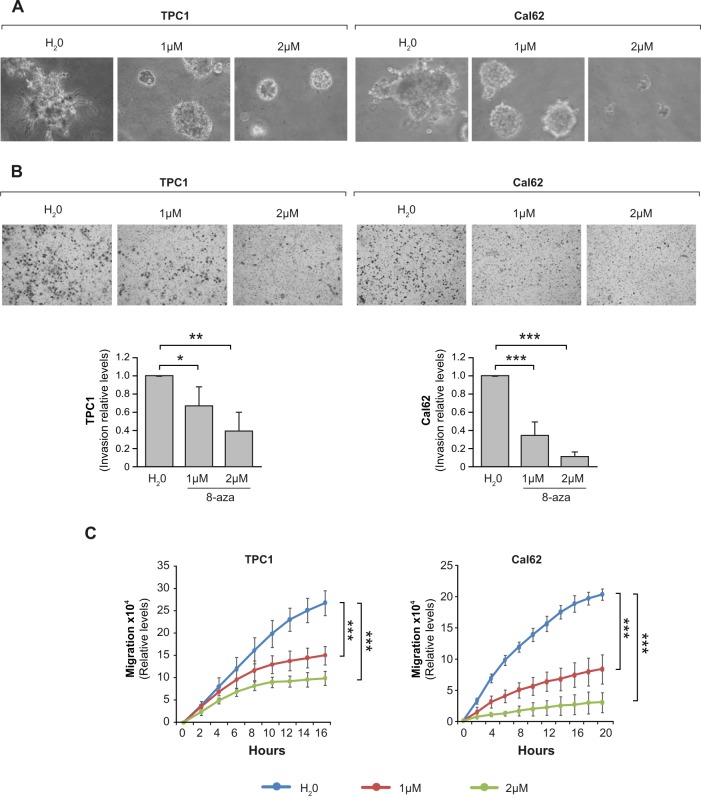
Inhibition of RNA A-to-I editing reduces 3D growth and 2D invasion and migration in thyroid cancer cells. TPC1 and Cal62 cell lines were treated with 8-azaadenosine (8-aza) (1 or 2 µM) or water (H_2_O). **a** 3D cell culture in Matrigel. **b** Upper panel: representative images of the lower chamber (invading cells). Bottom panel: cell invasion relative to the nontreated cells. **c** Quantification from a wound-healing assay at the indicated time points after scratching. Values represent mean ± SD (*n* = 3). *p < 0.05; **p < 0.01; ***p < 0.001.

Our results thus far implicate ADAR1-mediated A-to-I editing in thyroid cancer progression, suggesting that the repression of this process might be therapeutically useful. To test this, we used 8-azaadenosine (8-aza) as an A-to-I editing inhibitor, in TPC1 and Cal62 cells. Consistent with its activity against other tumor types [39], 8-aza inhibited the editing activity of both cell lines at a concentration of 1 and 2 µM, which were optimal in our model (Fig. 6a). By contrast, *ADAR1* mRNA levels remained stable in both cells lines (Fig. 6b), demonstrating that 8-aza targets ADAR activity only. Mirroring the effects of *ADAR1* silencing, 8-aza decreased cell viability/proliferation in a dose-dependent manner, as measured by MTT reduction and crystal violet staining (Fig. 6c, d). To better assess cell growth, we used the 3D Matrigel model, and confirmed that cell growth was impaired in the presence of different concentrations of 8-aza (Fig. 7a). Lastly, 8-aza impeded invasion (Fig. 7b) and migration (Fig. 7c) of TPC1 and Cal62 cells. Overall, our data highlight the A-to-I RNA editing pathway as an important mechanism for thyroid cancer progression and a possible new target for thyroid cancer therapy.

## Discussion

A better understanding of new mechanisms in tumorigenesis is crucial to develop treatment strategies for cancer therapy. RNA editing is frequently upregulated in cancer, and has the capacity to impact RNA stability and splicing, protein sequence, and miRNA–target interactions. One of the most studied RNA editing phenomena is the conversion of adenosine to inosine involving the RNA editing enzyme ADAR1 [40–42]. Indeed, ADAR1-mediated RNA editing has emerged as a dominant driver of cancer relapse and progression [43–45], and *ADAR1* is a demonstrated oncogene in other tumor types, including oral cancer [46], cervical cancer HeLa cells [47], lung cancer [48], and esophageal squamous cell carcinoma [45]. However, the functional impact of ADAR1 expression and the underlying mechanisms remain largely unclear.

The present study expands our knowledge of RNA editing in thyroid cancer, the most common endocrine malignancy with a steadily-rising incidence [17]. Analysis of TCGA data [19] shows that A-to-I editing is significantly elevated in thyroid tumors [13, 14], and *ADAR1* expression correlates with a worse progression-free survival [38]. We confirm these data and describe for the first time, to our knowledge, that ADAR1 editing activity has an important role in thyroid cell tumorigenesis, as loss of ADAR1 function profoundly suppresses proliferation, invasion, and migration both in 2D and 3D cultures. The fact that the same results were obtained in 3D cultures is encouraging as these systems have proven to be more similar to the in vivo environment. Patient-derived tumor cells grow in a 3D conformation with a specific organization and architecture that 2D monolayer cell culture cannot replicate, and it is known that cells grown in a 3D model are physiologically more relevant and better recapitulate some biological features such as cell viability and proliferation [22].

In addition, we confirm the in vivo function of *ADAR1* in a mouse xenograft model. Thus, both the 3D in vitro and in vivo systems are useful platforms for future studies to screen for molecules, such as 8-aza, that inhibit ADAR1 editing activity. We demonstrate that the treatment of thyroid tumor cells with 8-aza reduces cell viability, growth, migration and invasion, and this effect occurs both in 2D and 3D systems. The use of 8-aza as a treatment for thyroid and other cancers is thus an attractive option. However, practically, this is tempered by the finding that 8-aza has significant hemodynamic effects and some adverse reactions when given to rats [49] and, consequently, no data exist on 8-aza treatment in animal cancer models. Nevertheless, our data propose RNA editing as a potential therapeutic target for thyroid cancer and open the possibility to design new molecules against ADAR1 activity with fewer adverse effects but with good therapeutic potential.

Interestingly, our data point to ZEB1, a master EMT transcription factor, as a key effector in the ADAR1 pathway in thyroid tumorogenesis. While A-to-I activity is elevated in thyroid cancer and other tumor types compared with normal tissues, in others are low. This divergent activity or expression of the editing enzymes in different tumors is not clearly understood (reviewed in [15]), but might explain the deregulation of noncoding RNA editing in different tumor types, which has been suggested to promote tumorigenesis. Because miRNA function strongly depends on its sequence complementarity with target genes, RNA editing can influence regulatory functions by decreasing the effective amount of WT miRNAs and generating novel miRNAs that inhibit a different set of targets. For instance, increased ADAR-mediated RNA editing of stem cell regulatory *let-7* miRNAs may enhance leukemic self-renewal and contribute to chronic myeloid leukemia [39]. By contrast, decreased RNA editing of miR-455-5p in melanoma allows for downregulation of tumor suppressor CPEB1 and promotes tumor progression [50]. In addition, ADAR1 is frequently downregulated in metastatic melanoma, where it controls the expression of ~100 miRNAs that regulate genes related to melanoma phenotypes [51]. In the context of thyroid cancer, we focused on an RNA-editing event in the seed region of the tumor suppressor miR-200b, a key regulator of EMT and cancer metastasis. miR-200b is one of the main regulators of ZEB1 and shows high levels of editing in TCGA PTC samples (*n* = 518) when compared with matched normal tissues (*n* = 69) [38]. We confirmed this phenomenon by analyzing the edited and WT miR-200b levels in six PTC patients, finding a significant increase in the edited/WT ratio in tumor samples. In addition, based on TCGA data, miR-200b editing levels correlate with a worse progression-free survival [38]. We found that edited miR-200b exhibits a lower activity against its target *ZEB1*-3′UTR in thyroid cancer cells, likely explaining the decrease in ZEB1 expression in *ADAR1*-silenced cells. Moreover, the impaired ability of edited miR-200b to inhibit ZEB1 could promote motility and invasion by reducing the total amount of WT miR-200b in the cell. Although we have focused our research on ZEB1 as a main target of miR-200b, this miRNA is a critical regulator for several processes, suggesting that similar effects might occur with other miR-200b targets—some of them crucial for important processes such as angiogenesis, apoptosis, cell cycle, invasion, metastasis, and chemosensitivity [52]. Our data suggest that inhibition of miR-200b editing is an additional strategy to decrease cancer cell aggressiveness and potentially resensitize cancer cells to chemotherapeutic agents. miR-200b is downregulated in ATC but not in PTC. Therefore, the mechanism of miR-200b inactivation in PTC is not its downregulation, but rather a higher editing rate elicited by ADAR1. By contrast, in ATC both processes could be involved simultaneously.

Thyroid cancer comprises different entities that range from differentiated tumors such as FTC and PTC to poorly differentiated (PDTC) and fully undifferentiated (ATC) tumors. It is well recognized that tumor suppressors and oncogenes are involved in the evolution of thyroid carcinomas. BRAF and RAS, which exert their effects *via* the differential activation of MAPK and/or PI3K signaling, are the driver oncogenes in this tumor type. Using specific kinase inhibitors, we show that ADAR1 regulation in thyroid cancer cells is PI3K-dependent; however, the simultaneous inhibition of both pathways promotes a more robust repression of ADAR1 than PI3K inhibition alone. Our data also indicate that the RAS oncogene, but likely not BRAF, modulates ADAR1 activity. Consistent with these data, the hyperactivation of signaling pathways in tumorigenesis as inducers of ADAR1 expression has been reported in other systems, such as in leukemia stem cells where JAK2 activates ADAR1 [39].

Our study shows that the regulation of the two isoforms of ADAR1, p150 and p110, by PI3K is different, as the activation of this pathway seems to increase the levels of p150 but not p110. This suggests that the two isoforms exhibit distinct functions and regulation, which could be explained by the fact that cytoplasmic ADAR1 p150 is interferon inducible whereas nuclear ADAR1 p110 is constitutively expressed [7]. The specific roles of these isoforms are unknown at present and future studies are needed to understand the role of each of these isoforms in thyroid cancer. Curiously, it has been described that the interaction between ADAR1 p150 and DICER in viral myocarditis promotes the expression of specific miRNAs [53]. As we have previously demonstrated the important role of miR-146b in thyroid cancer as an oncogenic miRNA that inhibits PTEN [10] and DICER1 [54], the aforementioned interaction could be tested in our system to question whether the ADAR1-DICER1 complex promotes the activity of this miRNA in thyroid cancer.

In summary, ADAR1 activation in cancer is a novel mechanism of tumorigenesis that merits continued in-depth study. ADAR1 has also been proposed as a new target for immune-oncology therapy, and it has recently been reported that silencing *ADAR1* could make tumor cells more susceptible to immunotherapy [48, 55, 56]. Our novel data establish for the first time the significant role of ADAR1-dependent editing in thyroid cancer, and provide insight into the mechanism of action of this process in thyroid tumorigenesis by unveiling miR-200b editing as responsible, at least in part, for the aggressive features induced by ADAR1. Finally, our results underscore RNA editing inhibition as a viable option for the treatment of advanced thyroid cancer.

## Materials and methods

### Patients

Samples of PTC tumors and contralateral normal thyroid tissue from the same patients (*n* = 6) were collected at the Biobank of the La Paz University Hospital (Madrid, Spain). The main clinical characteristics of the patients are summarized in Table S1. Informed consent was obtained from all the patients following the protocols approved by hospital ethics committee.

### Cell culture

The thyroid cell lines TPC1, Cal62, and 8505c were grown in Dulbecco’s modified Eagle’s medium (DMEM) supplemented with 10% fetal bovine serum (FBS). The sources of the cell lines have been described [57]. PCCl3-BRAF and PCCl3-HRAS were derived from PCCl3 rat thyroid follicular cells [58], to obtain doxycycline-inducible expression of BRAF^V600E^ or HRAS^V12^ [57, 59, 60]. All cell lines used in this work were tested for mycoplasma contamination and authenticated every 6 months by short tandem repeat profiles using the Applied Biosystems Identifier kit in the Genomic Facility at the Institute of Biomedical Research (IIBm; Madrid, Spain).

### Transfections

siRNAs and miRNA mimics were transfected using Lipofectamine RNAimax (Thermo Fisher). Luciferase assays were performed using Lipofectamine 2000 (Thermo Fisher). OptiMEM was used as a reduced serum medium in all transfections.

### siRNAs, mimics, and plasmids

The siRNAs were purchased from Thermo Fisher (siControl [Silencer Select Negative Control #1 4390843] siADAR1 #1 [4390824] and siADAR1 #2 [AM5133]). miR-200b-3p mimics for the WT (C-300582-07-0002 miRIDIAN miRNA) and the edited form (mature sequence: UAAUGCUGCCUGGUAAUGAUGA) and the negative control (miRIDIAN miRNA Mimic Negative Control #1) were purchased from Cultek. In addition, the following vectors were obtained from Addgene: pCI-neo-RL-ZEB1 (Addgene plasmid #35535) and pCI-neo-RL-ZEB1 200bmut × 5 (Addgene plasmid #35537) [30], and were gifts from Greg Goodall (University of South Australia). A vector expressing luciferase and GFP, CMV-Firefly Luc-IRES-EGFP, was constructed by Dr J. Blanco (IQAC-CSIC), and Cal62 human tumoral thyroid cells stably expressing the vector (Cal62-Luc) were generated by Dr Eugenia Mato (IIB, Sant Pau).

### RNA quantification

Total RNA was isolated from cells with Trizol Reagent (Invitrogen). Reverse transcription-PCR (RT-PCR) assays for coding genes was performed using the M-MLV Reverse Transcriptase Kit (Promega Corporation). Quantitative RT-PCR (qRT-PCR) was performed with Kapa Sybr Fast Universal Kit from Sigma-Aldrich. All primers were purchased from Sigma-Aldrich and are described in Table S2.

### RNA editing quantification

To quantify RNA editing activity, we analyzed the previously described editing site in the *AZIN1* transcript. We used the RNA editing site-specific primer design strategy that is compatible with SYBR green qRT-PCR protocols (RESSq-PCR), as described [61]. Briefly, one pair of RESSq-PCR primers detects the WT transcript (an “A” base), and the other pair detects the edited transcript containing a “G” base, representing the inosine substitution. To enhance allelic discrimination, the primers differ in the 3’ nucleotide position to detect the WT A or the edited G nucleotide, and an additional mismatch was incorporated two nucleotides upstream of the 3′ primer end. Both pairs of primers are described in Table S2. For miR-200b WT (UAAUACUGCCUGGUAAUGAUGA) and edited variant (UAAUGCUGCCUGGU AAUGAUGA) recognition, miRNAs were retrotranscribed using the miRCURY™ LNA™ Universal RT miRNA PCR Kit, and qRT-PCR was performed using the miRCURY LNA SYBR^®^ Green PCR Kit (Qiagen) with specific commercial primers for WT miR-200b WT and edited miR-200b, optimized with LNA technology to enable sensitive and specific miRNA quantification.

### Protein extraction, western blotting, and antibodies

Cells were lysed and proteins extracted with RIPA buffer [10, 54]. Protein concentration was measured using the Bradford method (Bio-Rad Laboratories). Samples were separated by SDS-PAGE and transferred to nitrocellulose membranes (Bio-Rad Laboratories). The following antibodies were obtained from Santa Cruz Biotechnology Inc.: ADAR1 (SC-73408), p-AKT ser473 (sc-7985-R), AKT (sc-8312), pERK Tyr204 (sc-7383), and HRAS (C-20) (sc-520). ERK1/2 (#9102) and ZEB1 (#3396) antibodies were purchased from Cell Signaling Technology Inc., the PCNA (ab92552) antibody was from Abcam and the GAPDH (MAB374) antibody was from EMD Millipore Corp.

### MTT assay and crystal violet-stained colonies

Thiazolyl blue tetrazolium bromide (MTT) assays were performed using a kit from Sigma-Aldrich (M2128-500MG). Cells were seeded in 96-well plates (1000 and 2000 cells/well for Cal62 and 8505c cell lines or 500 and 1000 cells/well for the TPC1 cell line) and measurements were carried out in a spectrophotometer. To assay cell viability by crystal violet staining, 2500 Cal62 or 8505c cells, or 1500 TPC1 cells were seeded in each well of a six-well plate 24–48 h after transfection. Individual wells were fixed in 4% formaldehyde after 7–10 days and stained with crystal violet. After extensive washing and drying, crystal violet was resolubilized in 1% acetic acid and quantified at 600 nm as an indirect measure of cell number.

### Three-dimensional cell growth

A total of 5000 Cal62 or TPC1 cells were resuspended in DMEM containing 5% Matrigel (Matrigel^®^ Growth Factor Reduced Basement Membrane Matrix [LDEV-free] from Corning, and were seeded on the top of a 24-well plate containing 500 μL of Matrigel, which was added 30 min before seeding. The medium was replaced every 3–4 days.

### Invasion and migration assays

Cell invasion was examined in Transwell cell culture chamber filters coated on the upper side with Matrigel (Corning Biocoat) as described [54]. Cells were seeded at 5 × 10^4^ for Cal62 and 8505c cells and 3 × 10^4^ for TPC1 cells in DMEM culture medium with 0.2% FBS 48 h after transfection. Wound-healing assays were performed as described [54], 48 h after transfection.

### In vivo study

Animal experimentation was performed in compliance with the European Community Law (86/609/EEC) and the Spanish law (R.D. 1201/2005), with the approval of the ethics committee of the Consejo Superior de Investigaciones Científicas (CSIC, Spain). Xenotransplants were established in 6-week-old female BALB/c nude (nu/nu) mice by subcutaneous injection in the left flank of 1 × 10^6^ Cal62-luc cells previously transfected with the corresponding siRNA and suspended in 50 μl of PBS mixed with 50 μl of Matrigel. Mice were randomly divided in three groups and xenografts were established in 22 animals. Tumor bioluminescent signals were determined in vivo at the indicated time points to calculate tumor growth, as described [10], and mice were imaged with the IVIS-Lumina II Imaging System (Caliper Life Sciences).

### Luciferase assay

To measure the reporter, *Renilla* luciferase, activity of the *ZEB1* 3′UTR, the pCI-neo-RL-ZEB1 vector (ZEB1 3′UTR) or the pCI-neo-RL-ZEB1 200bmutx5 vector, containing mutations in the miR-200b-3p binding sites (*ZEB1* 3′UTR miR-200b-mut) was transfected together with a control Firefly luciferase plasmid and the corresponding siRNA or mimic. At 48 h after transfection, cells were harvested and assayed with the Dual-Luciferase Reporter Assay Kit (Promega Corporation). *Renilla* luciferase activity was normalized to firefly luciferase activity.

### Cell treatments with AKTi, selumetinib, and 8-azaadenosine

Cells were treated with AKTi (Tocris), selumetinib (LC Laboratories), or a combination of both drugs for 24 h to final concentrations of 10 and 50 µM, respectively. Both drugs were dissolved in DMSO. 8-azaadenosine (8-aza) (AKOS030530872, Akos Consulting & Solutions GmbH) was used to inhibit RNA editing. 8-aza dissolved in water was added to the cell culture medium at the indicated final concentrations.

### Statistical analysis

Results are expressed as the mean ± SD of at least three different experiments performed in triplicate. Results from the in vivo studies and patient analysis are expressed as the mean ± SEM. Statistical significance was determined by Student’s *t* test analysis (two tailed) and differences were considered significant at a *P* value < 0.05.

## Supplementary information

Suppl_Table 1

Suppl_Table2

Figure Suppl-1

Figure Suppl-2

Suppl_ Figure Legends
