# Supplementary material for: ADAR1-mediated RNA editing is a novel oncogenic process in thyroid cancer and regulates miR-200 activity
Source: Oncogene. 2020 Mar 10;39(18):3738–53. doi: 10.1038/s41388-020-1248-x (PMC7190574; doi:10.1038/s41388-020-1248-x)
Supplement: Supplementary file 1 — Suppl_Table 1 [file 41388_2020_1248_MOESM1_ESM.docx]

**TABLE S1**. Patients clinic-pathological characteristics.

| **Patient ID** | **Gender** | **Age at diagnosis** | **Diagnosis** | **TNM** | **Extrathyroidal extension** | **Vascular invasion** | **Total thyroidectomy** |
| --- | --- | --- | --- | --- | --- | --- | --- |
| 1 | M | 67 | PTC Classic | T2N1aM0 | NO | NO | YES |
| 2 | M | 40 | PTC Classic | T1bN1aM0 | NO | NO | YES |
| 3 | M | 50 | PTC Classic | T1bNxM0 | NO | NO | YES |
| 4 | F | 33 | PTC Follicular Variant | T1bNxM0 | NO | NO | YES |
| 5 | F | 44 | PTC Classic | T1aNxM0 | NO | NO | YES |
| 6 | M | 28 | PTC Classic | T3N1bM0 | YES | YES | YES |
