# Supplementary material for: ADAR1-mediated RNA editing is a novel oncogenic process in thyroid cancer and regulates miR-200 activity
Source: Oncogene. 2020 Mar 10;39(18):3738–53. doi: 10.1038/s41388-020-1248-x (PMC7190574; doi:10.1038/s41388-020-1248-x)
Supplement: Supplementary file 2 — Suppl_Table2 [file 41388_2020_1248_MOESM2_ESM.docx]

**TABLE S2**. Primers.

| **GENE** | **FORWARD PRIMER** | **REVERSE PRIMER** |
| --- | --- | --- |
| ADAR1 | CATCAGCGGGCTGTTAGAAT | CTTGGCCACTTTCTTGCTTC |
| AZIN1 WT | CATTCAGCTCAGGAAGAAGACATCT | AATACAAGGAAGATGAGCCTCTGTTTAC |
| AZIN1 Edited | ACTGAATGACATCATGTAATAAATGGCT | GAGCTTGATCAAATTGTGGCAG |
| GAPDH | TGCACCACCAACTGCTTAGC | GGCATGGACTGTGGTCATGAG |
