# Supplementary figures and images for: ADAR1-mediated RNA editing is a novel oncogenic process in thyroid cancer and regulates miR-200 activity
Source: Oncogene. 2020 Mar 10;39(18):3738–53. doi: 10.1038/s41388-020-1248-x (PMC7190574; doi:10.1038/s41388-020-1248-x)

**A**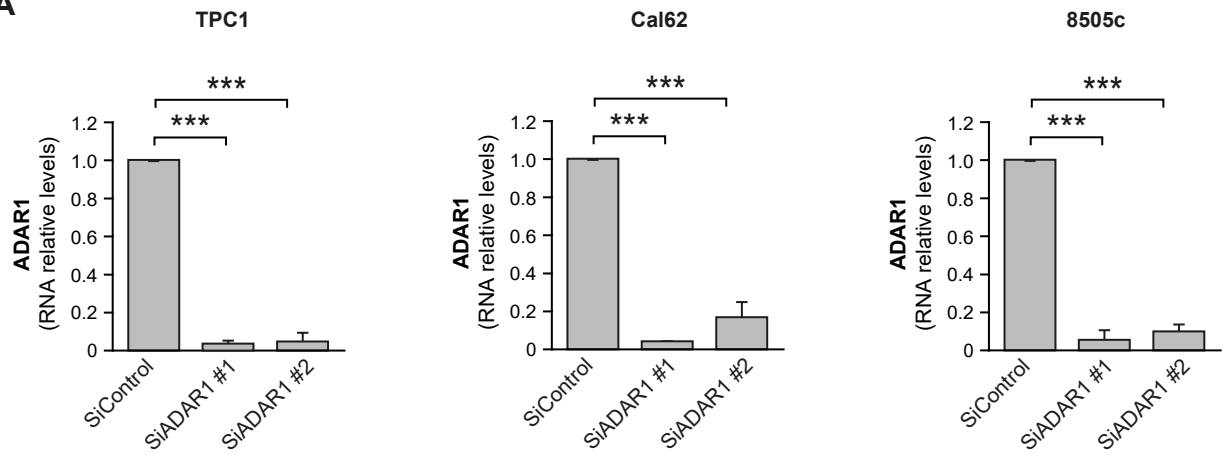**B**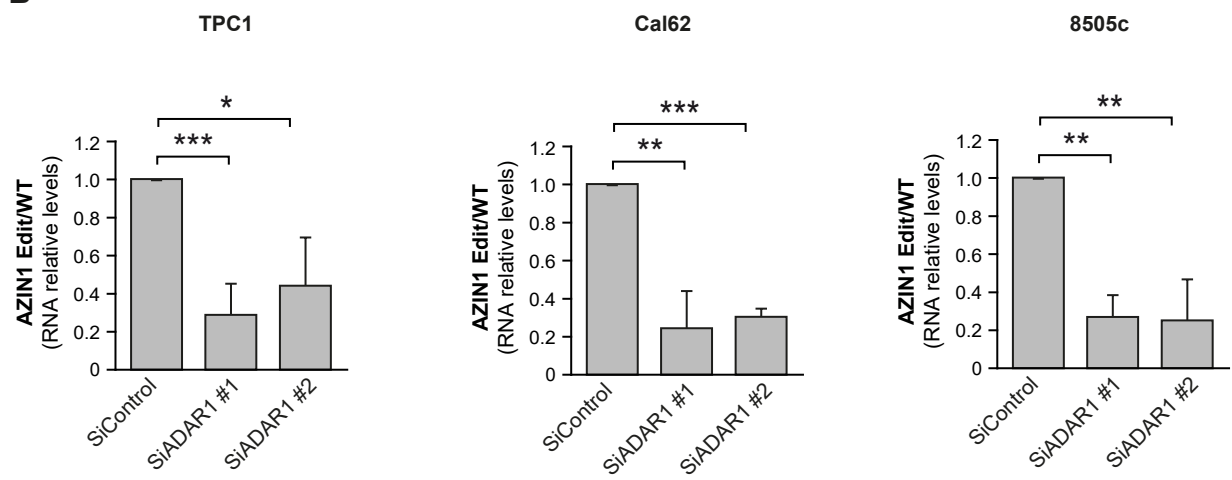

Supplement: Supplementary file 3 — Figure Suppl-1 [file 41388_2020_1248_MOESM3_ESM.pdf]

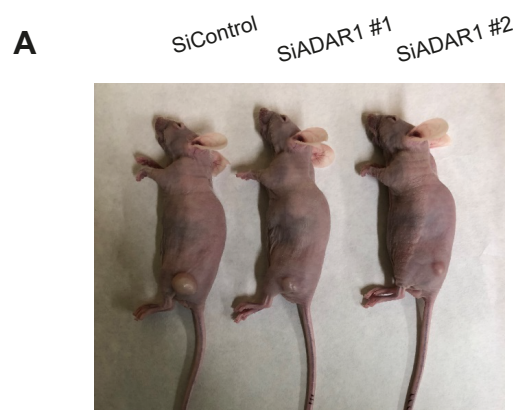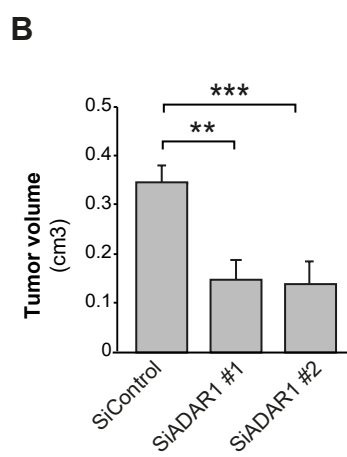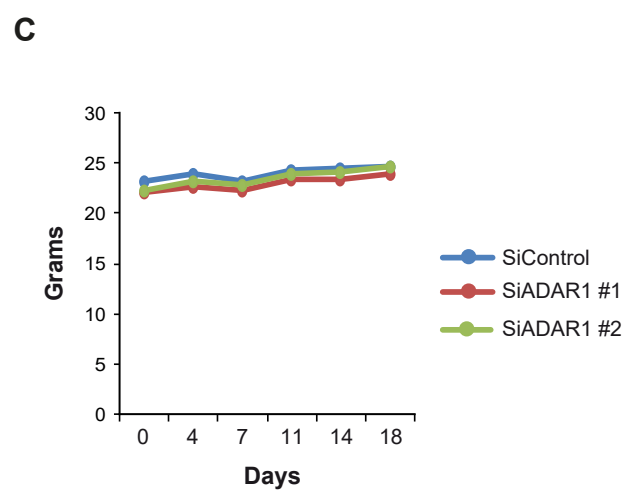

Supplement: Supplementary file 4 — Figure Suppl-2 [file 41388_2020_1248_MOESM4_ESM.pdf]
