# Supplementary material for: ADAR1-mediated RNA editing is a novel oncogenic process in thyroid cancer and regulates miR-200 activity
Source: Oncogene. 2020 Mar 10;39(18):3738–53. doi: 10.1038/s41388-020-1248-x (PMC7190574; doi:10.1038/s41388-020-1248-x)
Supplement: Supplementary file 5 — Suppl_ Figure Legends [file 41388_2020_1248_MOESM5_ESM.docx]

**Supplementary Figure Legends**

**Supplementary Figure 1**. TPC1, Cal62, and 8505 cell lines were transfected with two different siRNAs against *ADAR1* (siADAR1 #1 and siADAR1 #2) or a control siRNA (siControl). (A) Relative *ADAR1* mRNA level assayed by qRT-PCR 72 h post-transfection. (B) Relative *AZIN1* Edited / *AZIN1* wild-type (*AZIN1* Edit/WT) mRNA levels assayed by RESS-qRT-PCR 72 h post-transfection. Values represent mean ± SD (*n* = 3). *p<0.05; **p<0.01; ***p<0.001.

**Supplementary Figure 2.** Xenograft tumors were generated by Cal62-Luc siControl (n=8), siADAR1 #1 (n=7) or siADAR1 #2 (n=7) subcutaneous injection. (A) Representative image of the endpoint (day 18) generated tumors. (B) Tumor volume relative to siControl tumors measured with a caliper at day 18. (C) Mouse weight at the indicated time points. Values represent mean ± SD (*n* = 3). **p<0.01; ***p<0.001.
